# Interaction of Norsecurinine-Type Oligomeric Alkaloids with α-Tubulin: A Molecular Docking Study

**DOI:** 10.3390/plants13091269

**Published:** 2024-05-03

**Authors:** Gérard Vergoten, Christian Bailly

**Affiliations:** 1 U1286—INFINITE, Lille Inflammation Research International Center, Institut de Chimie Pharmaceutique Albert Lespagnol (ICPAL), Faculté de Pharmacie, University of Lille, 3 rue du Professeur Laguesse, 59006 Lille, France; 2 CNRS, Inserm, CHU Lille, UMR9020-U1277-CANTHER—Cancer Heterogeneity Plasticity and Resistance to Therapies, OncoLille Institut, University of Lille, 59000 Lille, France; 3 Institute of Pharmaceutical Chemistry Albert Lespagnol (ICPAL), Faculty of Pharmacy, University of Lille, 59006 Lille, France; 4 OncoWitan, Scientific Consulting Office, 59290 Lille, France

**Keywords:** anticancer agents, oligomeric alkaloids, *Flueggea virosa*, molecular docking, norsecurinine, *Securinega* alkaloid, tubulin binding

## Abstract

The medicinal plant *Securinega virosa* (Roxb ex. Willd) Baill., also known as *Flueggea virosa* (Roxb. ex Willd.) Royle, is commonly used in traditional medicine in Africa and Asia for the management of diverse pathologies, such as parasite infections, diabetes, and gastrointestinal diseases. Numerous alkaloids have been isolated from the twigs and leaves of the plant, notably a variety of oligomeric indolizidine alkaloids derived from the monomers securinine and norsecurinine which both display anticancer properties. The recent discovery that securinine can bind to tubulin and inhibit microtubule assembly prompted us to investigate the potential binding of two series of alkaloids, fluevirosines A–H and fluevirosinine A–J, with the tubulin dimer by means of molecular modeling. These natural products are rare high-order alkaloids with tri-, tetra-, and pentameric norsecurinine motifs. Despite their large size (up to 2500 Å^3^), these alkaloids can bind easily to the large drug-binding cavity (about 4800 Å^3^) on α-tubulin facing the β-tubulin unit. The molecular docking analysis suggests that these hydrophobic macro-alkaloids can form stable complexes with α/β-tubulin. The tubulin-binding capacity varies depending on the alkaloid size and structure. Structure-binding relationships are discussed. The docking analysis identifies the trimer fluevirosine D, tetramer fluevirosinine D, and pentamer fluevirosinine H as the most interesting tubulin ligands in the series. This study is the first to propose a molecular target for these atypical oligomeric *Securinega* alkaloids.

## 1. Introduction

Oligomeric natural products (NPs) are not excessively frequent in nature. Tri- and tetrameric NPs can be found but they are not largely represented, in contrast to dimeric NPs which are widespread in plants, microorganisms, and marine bryozoa. Bis-diterpenoids have been known for a long time and can form a large variety of homo- and hetero-dimers [1]. A large diversity of dimeric compounds can be found, including methylene-bridged dimers formed through nonenzymatic dimerization [2]. Occasionally, one can discover trimeric and tetrameric compounds, notably oligomeric sesquiterpenoids [3,4]. Tetrameric terpenoids are quite rare, in contrast to tetrameric proanthocyanidins regularly discovered. For example, a recent study with lotus seeds (*Nelumbo nucifera* Gaertn.) has identified 16 dimeric, 18 trimeric, and 4 tetrameric proanthocyanidins [5]. Certain oligomeric proanthocyanidins display useful bioactivities, such cinnamon polyphenols characterized as antidiabetic agents [6]. There are also phloroglucinol dimers and trimers acting as acetylcholinesterase inhibitors with antibacterial properties [7], and ellagitannin trimers such as rugosin G, which is a more potent inhibitor of human histidine decarboxylase than related ellagitannin monomers and dimers [8]. Various catechin oligomers have been discovered, including complex products with a high molecular weight, such as the antimicrobial ellagitannin sanguiin H-6 (C_82_H_54_O_52_, Mw: 1871.3 g/mol) active against methicillin-resistant *Staphylococcus aureus* (MRSA) [9,10]. Oligomeric polyphenols can be found in plants, such as catechin oligomers identified in black tea [11,12] and resveratrol tetramer and pentamer found in Dipterocarpaceaeous plants [13]. Resveratrol hexamer and even an octamer (upunaphenol Q) have been identified [14,15].

**Figure 1:**
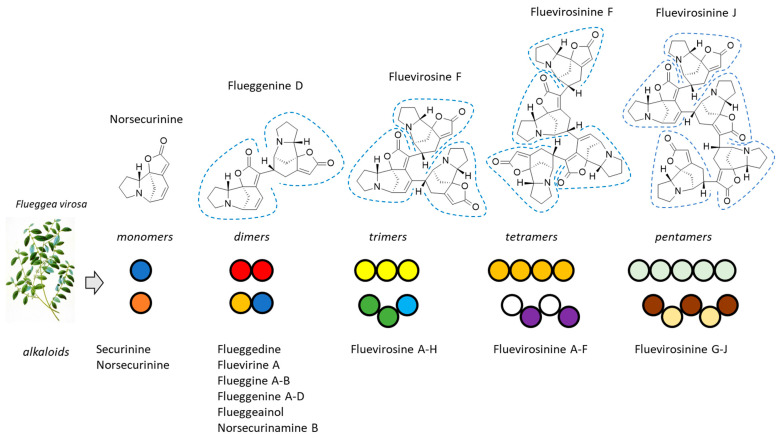
Oligomeric alkaloids from *Flueggea virosa*. Numerous alkaloids have been isolated from the plants, including a variety of monomeric, dimeric, trimeric, tetrameric, and pentameric alkaloids. Representative products are illustrated, with the norsecurinine units circled.

Oligomeric alkaloids are even rarer than oligomeric terpenoids. Trimeric alkaloids are known, such as trisindoline, comprising an isatin core with two indole moieties [16], and divers citrinin trimers (tricitrinols A–C and neotricitrinols A–C), isolated from fungi [17,18]. But in general, alkaloids rarely form oligomeric structures in nature. They can be synthesized (e.g., terguride oligomers and noacronycine oligomers) [19,20,21], but they are essentially monomeric or dimeric in nature. However, there exists an exception with the *Securinega* alkaloids which are indolizidine alkaloids extracted from several *Securinega* species [22]. The two main species are *S. suffruticosa* (Pall.) Rehd. and *S. virosa* (Roxb ex. Willd) Baill., also known as *Flueggea suffruticosa* (Pall.) Baill. and *F. virosa* (Roxb. ex Willd.) Royle, respectively [23]. The family includes over 65 monomeric and oligomeric alkaloids, many of which display interesting anti-inflammatory and anti-proliferative activities [22]. They derive from the monomer securinine and norsecurinine which are precursors for the formation of dimers such as flueggine B, but also trimers, tetramers, and pentamers [24]. These oligomeric alkaloids are unique in the plant kingdom (Figure 1).

These rare big alkaloids are intriguing. What are their molecular targets and mechanism of action? What is their function in cells and in the plants? Oligomeric alkaloids have been little studied thus far, although they have attracted a lot of attention from chemists interested in deciphering the oligomerization mechanism. Recently, the total synthesis of several dimeric *Securinega* alkaloids has been reported, such as flueggeacosine B and flueggenines D and I [25]. But the high-order alkaloids, notably the tetra- and pentamers, can be accessed only via plant extraction [24], and their mechanism of action remains little known in general.

**Figure 2:**
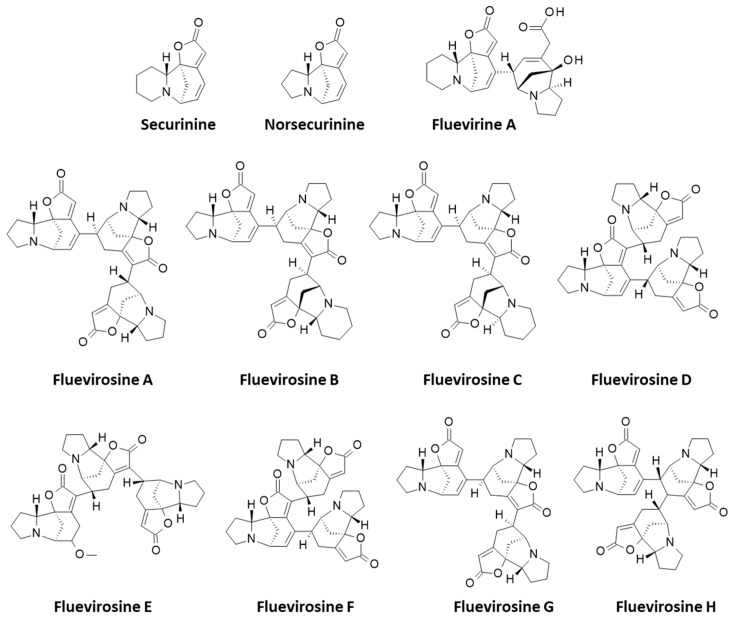

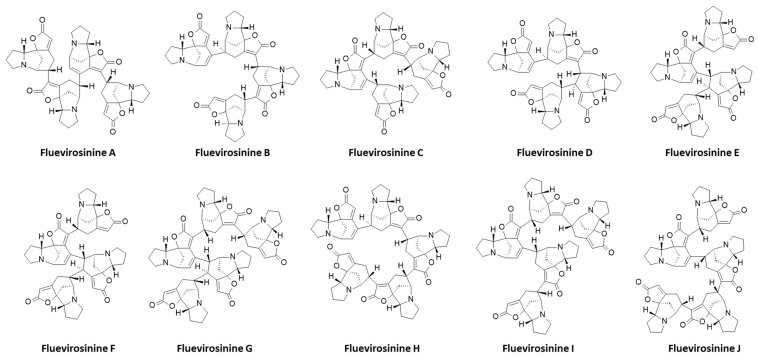
Structures of (nor)securinine (monomers), fluevirine A (dimer), and a series of trimeric alkaloids, fluevirosines A–H. Compound identity numbers: 102491901, 102491902, 102491903, 102432340, 129901232, and 129901233 for fluevirosines A–D and G–H, respectively. Fluevirosines E and F, not listed in the PubChem databank (https://pubchem.ncbi.nlm.nih.gov/, last accessed 10 April 2024), were not modeled. Structures of the tetrameric alkaloids, fluevirosinines A–F, and pentameric alkaloids, fluevirosinines G–J (compound identity numbers: 102492339 [A], 129901030 [B], 132602929 [C], 132602930 [D], 132602931 [E], 129901033 [G], 132602933 [H], 129904792 [I], and 129904804 [J]).

Recent studies have provided important information about the mechanism of action of the monomeric unit of these mega-alkaloids. Securinine has been shown to inhibit the proliferation of cancer cells by targeting the PI3K/Akt/mTOR kinase signaling pathway and the regulation of the JAK-STAT3 signaling pathway [26]. At the molecular level, securinine has been shown to induce mitotic block in cancer cells by binding to tubulin and inhibiting microtubule assembly [27]. The mechanism of action is consistent with previous observations showing that the alkaloid induced S-phase cell cycle and apoptosis [28]. These considerations prompted us to investigate the tubulin-binding capacity of *Securinega* alkaloids using a molecular docking approach. Our first study was centered on a series of dimeric alkaloids, fluevirines A–F and flueggenines A–I. The data revealed that two main compounds (fluevirine A and flueggenine I) were able to form stable complexes with α/β-tubulin dimers upon binding to the pironetin site of tubulin [29]. We extended the study to investigate the high-order alkaloids, including tri-, tetra-, and pentameric alkaloids, namely, fluevirosines A–H and fluevirosinines A–J (Figure 2). These oligomers have all been isolated from the twigs and leaves of *Flueggea virosa* [30,31,32]. The study suggests that the high-order alkaloids can also form stable complexes with tubulin dimers, with the macro-alkaloids trimer fluevirosine D, tetramer fluevirosinine D, and pentamer fluevirosinine H emerging as the most interesting tubulin ligands in the series. Structure–binding relationships are discussed.

## 2. Results

**Figure 3:**
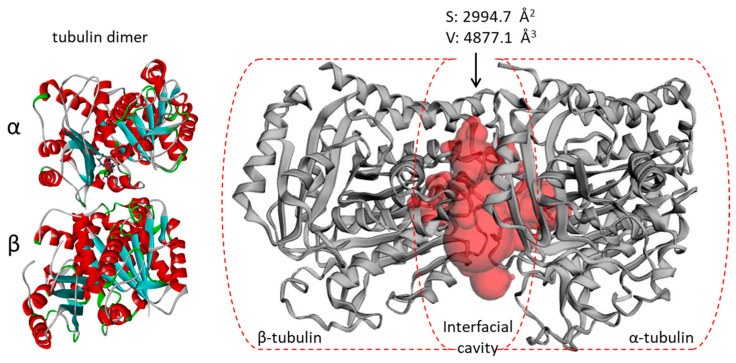
Binding site analysis of tubulin α/β using web server CASTp 3.0. (**left**) Molecular model of pironetin-bound tubulin dimer (from PDB: 5FNV). (**right**) The analysis of the tubulin dimer primarily reveals the large interfacial area at the junction of the α/β units (in red), with the indicated surface (S) and volume (V).

The crystallographic structure 5FNV corresponding to the α/β-tubulin dimer in interaction with the natural product pironetin was selected for the docking analysis [33]. This structure displays a large cavity between the two monomers, accessible to small molecules. We have recently shown that this structure, 5FNV, was better adapted than the other crystallographic structure, 1TVK, initially used for the docking of securinine to the α-tubulin unit [27]. 1TVK requires the removal of the GDP molecule present in the cavity prior to perform the docking [29]. With structure 5FNV, there is no need to alter the protein organization to allow the test ligand to bind to the interface between the α/β-tubulin monomers. The central cavity is large, with a size estimated at 1595 Å^3^ when the calculation is based on the protein van der Waal’s surface [34], and at 4877.1 Å^3^ when the molecular surface envelope is calculated using a dot surface numerical algorithm [35] (Figure 3).

The interfacial cavity is large enough to accommodate the alkaloids, even the bulky oligomers. The volumes of the tetrameric and pentameric alkaloids are about 2000 and 2500 Å^3^ (Table 1) compared to the volume of 4877.1 Å^3^ for the protein cavity (volumes measured with the same methodology). Therefore, the volume of the cavity is amply sufficient for a ligand to bind to a site into this pocket. The oligomeric alkaloids all present a large hydrophobic solvent accessible surface area (SASA), well superior to the hydrophilic SASA. They are adapted to bind to the hydrophobic binding pocket at the α/β-tubulin interface.

The docking analysis was initiated with the fluevirosine series of trimeric molecules. They are relatively large molecules (Mw > 600 g/mol), with a volume of about 1650 Å^3^ (Table 1). Each compound was docked into the tubulin structure (5FNV), with the compound initially positioned near the pironetin binding site, in the vicinity of the key cysteine residue (Cys316) to which pironetin can bind covalently [33]. For each compound, the empirical energy of interaction (ΔE) and free energy of hydration (ΔG) were calculated (Table 2). For the sake of convenience (to avoid an error of structure for these complex molecules), two compounds, fluevirosines E and F, were omitted because they are not listed in the PubChem databank. Molecular models for fluevirosines A–D and G–H were elaborated and the ΔE/ΔG values calculated (Table 2).

**Figure 4:**
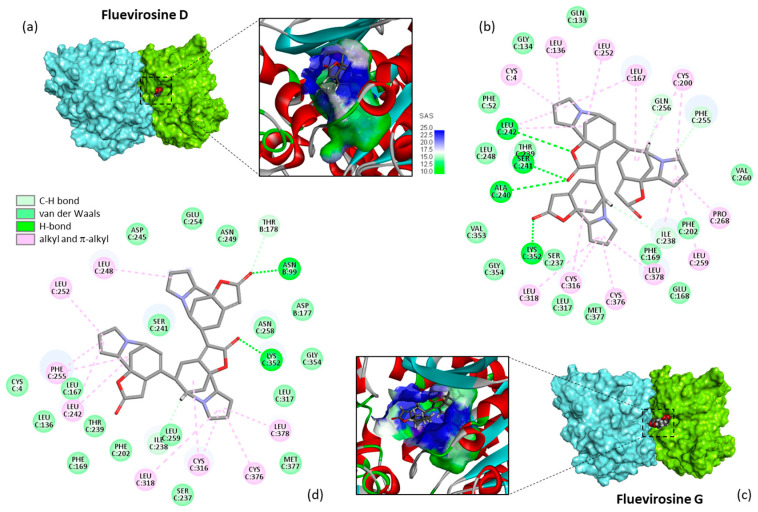
Molecular model of trimers, fluevirosines D and G, bound to tubulin. (**a**) A surface of fluevirosine D bound to the α/β-tubulin dimer, with a close-up view of the compound inserted into the binding cavity, with the solvent-accessible surface (SAS) surrounding the drug binding zone (color-code-indicated). (**b**) Binding map contact for fluevirosine D bound to α-tubulin (color-code-indicated). (**c**) A similar model for fluevirosine G with (**d**) the corresponding binding map contact.

In the series of trimers, the measured energies of interaction (ΔE) rank in the order fluevirosine D < G < H < A < B < C. The last two compounds, fluevirosines B–C, are structural isomers, not well adapted to bind to the tubulin site. In contrast, fluevirosines G–H (also isomers) are better suited for tubulin binding. But the best compound in this series is fluevirosine D, which gave a ΔE value clearly superior (more negative) than those determined with the other trimers and also with dimeric alkaloids such as fluevirine A, for example (ΔE = −73.9 and −94.5 kcal/mol for fluevirine A and fluevirosine D, respectively). Representative molecular models of fluevirosine D and G bound to the α/β-tubulin dimer are shown in Figure 4. Fluevirosine G displays multiple interactions with the tubulin dimer. The complex is stabilized by a range of van der Waals contacts and alkyl/π-alkyl interactions. It is interesting to note that there are two key H-bonds, one with Lys352 on the α-tubulin subunit and one with Asn99 on the β-tubulin subunit. The binding site goes from the top of the cavity nearby the Cys316 residue, up to the α/β-tubulin interface. The alkaloid binding configuration is a little distinct with fluevirosine D, which penetrates more deeply into the binding cavity and interacts exclusively with the α-tubulin subunit. In this case, there are four key H-bonds between the ligand and the protein (Ala240, Ser241, Leu242, and Lys352), in addition to a panoply of van der Waals contacts, C-H bonds, and alkyl/π-alkyl interactions. Fluevirosine D inserts its trimeric core more deeply into the cavity than fluevirosine G, and its protein anchorage is stronger. Fluevirosine D is the best trimeric alkaloid in the series. It coordinates with the triad Ser241-Leu242-Lys352 of α-tubulin through its central norsecurinine unit, without contacting the facing β-tubulin chain (Figure 4).

**Figure 5:**
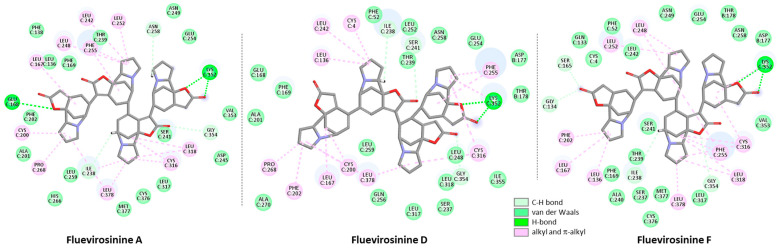
Binding map contact for tetrameric alkaloids, fluevirosinines A, D, and F.

Next, we investigated the six tetrameric alkaloids, fluevirosinines A–F. The calculated binding energies (ΔE in Table 2) pointed to two subgroups, one with fluevirosinines A, D, and F (ΔE < 100 kcal/mol) and the other one with fluevirosinines B, C, and E (ΔE > 100 kcal/mol). The best tetramer is fluevirosinine D and the one less prone to bind to tubulin is fluevirosinine E. The comparison of the binding models for fluevirosinines A, –D, and –F shows that the three compounds bind similarly to the tubulin dimer, with a common H-bond with residue Lys352 on the α-tubulin subunit (Figure 5). This is the essential ligand–protein contact, common to all molecules. This Lys352 residue is located at the entrance of the α-tubulin binding pocket, facing the β-unit of the tubulin dimer. There are more than 30 contacts between fluevirosinine F and α-tubulin, but only one contact with β-tubulin.

The stability of the alkaloid–protein complex is essentially maintained by a diversity of weak interactions, mostly van der Waals contacts. Nevertheless, the four units of the molecule participate in the interaction. Three of the four norsecurinine units of fluevirosinine F deeply penetrate the hydrophobic cavity, whereas the fourth is exposed to the outside of the groove at the α/β-tubulin junction, as illustrated in Figure 5. It is a large alkaloid (Mw = 813 g/mol and 2052 Å^3^) but well adapted to bind to α-tubulin.

**Figure 6:**
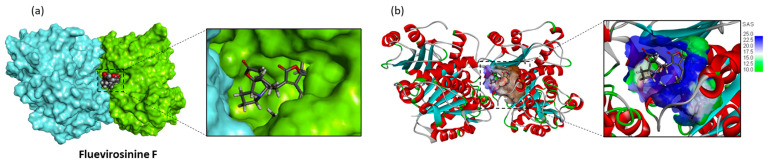
A model of fluevirosinine F (tetramer) bound to tubulin. (**a**) The α/β-tubulin dimer with the bound ligand, fluevirosinine F, to show its fifth (nor)securinine unit exposed at the surface of the α-tubulin unit (in green). (**b**) A ribbon model of the tubulin dimer, with a detailed view of the compound in the binding cavity. The solvent-accessible surface (SAS) surrounding the drug binding zone is color-code-indicated.

The binding of the pentameric alkaloids is similar to that observed with the tetramers. Fluevirosinines G and H were found to form more stable complexes with α/β-tubulin than fluevirosinines I and –J. A representative model of fluevirosinine H bound to the protein illustrates the occupation of the binding cavity by the compact alkaloid (Figure 6). The key H-bond with Lys352 remains present and 38 ligand–protein contacts stabilize the molecular edifice, including 35 with α-tubulin and 3 with β-tubulin. These oligomers all adopt a relatively compact, globular configuration to bind to the protein; they do not really present an extended conformation. The best ligand in the series is fluevirosinine G, and its binding process is very similar to that of fluevirosinine H (ΔE = −117.00 and −113.90 kcal/mol, respectively). Among the pentameric molecules, our favorite compound is fluevirosinine H, with a more favorable hydration free energy (ΔG = −45.10 kcal/mol) compared to fluevirosinine G (ΔG = −32.90 kcal/mol). Water interactions can make the difference; fluevirosinine H offers a good compromise between ΔE and ΔG values. The compound takes advantage of its five norsecurinine units to interact with tubulin. The large size of fluevirosinine H (Mw = 1016 g/mol and 2596 Å^3^) is apparently not an obstacle for tubulin binding, at least not from this docking perspective.

## 3. Discussion

These atypical oligomeric alkaloids were isolated from the twigs and leaves of *Flueggea virosa* about ten years ago [30,31,32]. They have been structurally characterized but little studied from a pharmacological viewpoint. A modest anti-HIV activity has been observed with fluevirosinine B (IC_50_ = 14.1 µM, compared to 0.12 µM with the reference antiretroviral drug nevirapine), whereas the other fluevirosinine derivatives and fluevirosines were less active or inactive. The second modestly active compound was fluevirosine G, with an IC_50_ of 58.7 µM [30]. They are present in the plant at a very low yield (0.00004% to 0.0003% for fluevirosinines A–C) [30], and only tiny amounts of the purified products were obtained (2–5 mg in general). The limited amount of product available limits the pharmacological investigations. The use of an in silico approach can help to define the mechanism of action of the compounds, at least to make a proposal for a potential target [36,37,38]. The recent characterization of the tubulin-binding property of securinine and its capacity to inhibit microtubule assembly [27] prompted us to investigate tubulin binding with the oligomeric derivatives. Interestingly, we found that these natural products are prone to tubulin binding. The large cavity at the interface of the α/β-tubulin dimer can accommodate a large alkaloid, even a tetra- or pentameric molecule.

An experimental validation will be required to support the in silico observation, but the docking simulation suggests that these oligomeric products can bind well to tubulin. We are aware of the limitation of the molecular docking simulation (often considered as inherently limited prediction), but the information is nevertheless useful to compare the different products and to identify novel chemotypes [39]. The docking analysis suggests that the oligomers can bind to the pironetin/colchicine site of α-tubulin. The best tubulin binder is the pentamer fluevirosinine H, which can insert deeply into the hydrophobic binding pocket. The compound uses each of its five units to interact with the protein, mostly via hydrophobic contacts.

**Figure 7:**
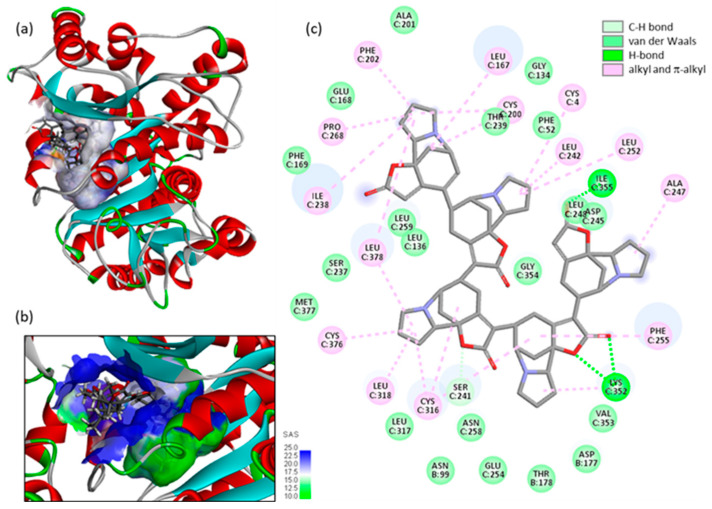
(**a**) A model of fluevirosinine F (pentamer) bound to the α-tubulin unit, with (**b**) the SAS area around the binding site and (**c**) the corresponding alkaloid binding map contact.

A common trait observed with the different compounds is the formation of a key H-bond with the Lys352 residue of α-tubulin. This contact is not entirely surprising as it has been observed with a variety of tubulin-biding small molecules, including oxadiazol-amine derivatives [40], triazolo-heterolignans [41], and benzimidazole derivatives [42]. This key lysine residue can engage in an H-bond with small molecules, or it can form a π–cation interaction with aromatics, as observed with the phenyl moiety of 1,4-naphthoquinone derivatives, for example [43]. Lys352 is one of the active sites for the interaction of the anticancer drug vinblastine with α,β-tubulin [44]. It was also initially considered as the covalent target point for pironetin [45], but a subsequent study demonstrated that, in fact, the product binds covalently to Cys316. The binding of pironetin to α-tubulin destabilizes the microtubule organization [33]. It is interesting to note that, in our model, fluevirosinine H binds to the same site, establishing contacts with both Lys352 and Cys316, in addition to many surrounding amino acids (Figure 7). It is an atypical bulky alkaloid, filling largely the interfacial cavity. The compound displays many H-bond acceptor atoms, but it interacts with the protein essentially via hydrophobic interactions, to position one of its norsecurinine in contact with the gating Lys352 residue. It seems to be well dimensioned for tubulin binding, and possibly to interfere with microtubule dynamics. The compound can be added to the long list of colchicine-site tubulin-binding alkaloids. This site is known to be is promiscuous, capable of accommodating a broad range of structurally distinct molecules that can vary in size, shape, and affinity [46]. Nevertheless, it is an attractive site for drug binding and drug design [47,48].

The modeling analysis suggests that all tri-, tetra-, and pentameric alkaloids can bind to tubulin. They are large alkaloids, comparable or superior in size to the vinca alkaloids. For example, the molecular weight of the tetramer fluevirosine D (C_48_H_50_N_4_O_8_, Mw = 811 g/mol) is identical to that of vinblastine (Mw = 811 g/mol). The pentamer fluevirosinine H is significantly bigger (C_60_H_65_N_5_O_10_, Mw = 1016.2 g/mol), but the binding cavity is deep enough to accommodate such a bulky compound. It is worth mentioning that there exists at least one large molecule known to bind to tubulin, a fullerene derivative C_60_(OH)_20_, which has been shown to bind to the same tubulin heterodimer but not to the same site [49]. If the prediction is correct, fluevirosinine H would be the largest alkaloid capable of binding to the colchicine/pironetin site. Hopefully, the docking analysis reported here will encourage experimental study to confirm the tubulin-binding capacity of these oligomeric alkaloids, to delimit the boundaries of the binding site, and to investigate their effects on microtubule dynamics. In the plant, the role of these endogenous oligomeric alkaloids found in the twigs and leaves is unknown at present. They may serve as defense against plant parasites and fungi, or to repel insects and herbivores.

## 4. Materials and Methods

### 4.1. Molecular Structures and Software

The tridimensional structures of the α/β-tubulin dimer were retrieved from the Protein Data Bank (PDB) (ID: 1TVK and 5FNV) [33,50]. The essential part of the docking study was performed with 5FNV (https://www.rcsb.org/structure/5FNV, last accessed 10 April 2024) which is a dimer with an overall structure resolution of 2.61 Å [34]. The molecular docking analysis was performed with the GOLD software (version 5.3, Cambridge Crystallographic Data Centre, Cambridge, UK). Prior to the docking operations, the structure of each ligand was optimized using a classical Monte Carlo conformational searching procedure via the BOSS software [51]. Molecular graphics and analysis were performed using Discovery Studio Visualizer, Biovia 2020 (Dassault Systèmes BIOVIA Discovery Studio Visualizer 2020, San Diego, Dassault Systèmes, 2020). The web server Computed Atlas of Surface Topography of proteins (CASTp) 3.0 was used to identify potential ligand-binding sites on the tubulin dimer. The molecular modeling software Chimera 1.15 was used for visualization [52].

### 4.2. In Silico Molecular Docking Procedure

The colchicine/pironetin binding area within the tubulin dimer structure (5FNV) was considered as the potential binding site for the studied alkaloids. During the process, the side chains of the following amino acids within the binding site were rendered fully flexible: Phe135, Ser165, Phe169, Cys200, Phe202, Ser241, Leu242, Phe255, Cys316, and Lys352. A docking grid centered in the volume defined by the central amino acid was defined based on shape complementarity and geometry considerations. In general, up to 100 poses considered as energetically reasonable were selected during the search for the correct binding mode of the ligand. The decision to select a trial pose was based on ranked poses, using the fitness scoring function (PLP score incorporated in GOLD v5.3) [53]. The same procedure was used to establish molecular models for all studied alkaloids.

In general, 6 poses were selected per analysis. The ranking led to the evaluation of the empirical potential energy of the interaction (ΔE), defined using the expression ΔE(interaction) = E(complex) − [E(protein) + E(ligand)]. The SPASIBA spectroscopic force field was used to calculate the final energy. The required parameters were derived from vibrational wavenumbers obtained in the infrared and Raman spectra of a large series of compounds of diverse chemical nature (organic molecules, amino acids, saccharides, nucleic acids, and lipids). The last step corresponded to a validation using the SPASIBA force field, an essential step to determine the best protein–ligand structure. This force field has been specifically developed to provide refined empirical MM force field parameters [54]. SPASIBA (integrated into CHARMM) empirical energies of interaction were calculated. This is an excellent system for reproducing crystal phase infrared data. SPASIBA has been specifically developed to provide refined empirical molecular mechanics force field parameters, as described in other studies [55,56,57]. Using this specific force field for Monte Carlo (MC) simulations achieved the same level of convergence as Molecular Dynamics (MD), with less computational time [58]. The Boss program and the Molecular Mechanics/Generalized Born Surface Area (MM/GBSA) procedure were used to evaluate free energies of hydration (ΔG), in relation with aqueous solubility [59].

## 5. Conclusions

Our computational study proposes a molecular target for the oligomeric alkaloids isolated from the medicinal plant *Securinega virosa* (syn: *Flueggea virosa*), which is used in China for the treatment of rheumatism, pruritus, and other ailments [60]. This plant contains a variety of high-molecular-weight alkaloids derived from the monomers securinine and norsecurinine, notably a range of rare tri-, tetra-, and pentameric alkaloids which have been little investigated thus far [30]. The monomeric and dimeric natural products can be accessed via total syntheses [25,61,62,63], but the access to the higher order alkaloids is very challenging via plant extraction procedures. Due to a lack of materials, the mechanism of action of the oligomeric alkaloids has rarely been investigated [22]. Computational approaches provide opportunities to apprehend their mechanism of action with the analysis of drug–target interactions. For the first time, a potential molecular target is proposed for the oligomeric alkaloids which can bind to the large interfacial cavity at the junction of α/β-tubulin dimers. Our docking study identifies the trimer fluevirosine D, tetramer fluevirosinine D, and pentamer fluevirosinine H as the best tubulin ligands among the two series of molecules tested (fluevirosines A–H and fluevirosinines A–J). The knowledge of a molecular target for these compounds may facilitate their extraction and isolation through bioguided procedures. This study shall encourage further exploration of the mechanism of action of these unusual oligomeric alkaloids.

## Figures and Tables

**Table 1:** Computed physico-chemical properties of selected alkaloids.

Molecule:	Norsecurinine	Flueggenine-I	Fluevirine-A	Fluevirosine-D	Fluevirosine-G	Fluevirosinine-A	Fluevirosinine-D	Fluevirosinine-F	Fluevirosinine-G	Fluevirosinine-H
Type	Monomer	Dimer	Dimer	Trimer	Trimer	Tetramer	Tetramer	Tetramer	Pentamer	Pentamer
Molecular Weight (g/mol)	203.2	438.5	424.5	609.7	609.7	813.0	811.0	813.0	1016.2	1016.2
Dipole moment (D)	6.0	12.5	4.3	5.4	4.0	7.4	11.2	14.1	7.8	14.5
Total SASA (Å^2^) ^a^	400.7	656.8	615.5	797.7	843.8	970.3	965.0	948.8	1158.7	1188.2
Hydrophobic SASA	210.1	543.1	401.7	588.1	610.7	715.9	680.5	718.9	832.1	893.3
Hydrophilic SASA	74.6	91.2	177.7	153.9	171.9	194.0	225.1	159.6	239.9	242.4
Molecular Volume (Å^3^)	668.4	1250.9	1193.7	1638.6	1679.7	2107.2	2074.8	2052.7	2546.4	2596.6
Donor H-bonds	0	0	2	0	0	0	0	0	0	0
Acceptor H-bonds	2	5	6	6	6	8	8	8	10	10
log P (octanol/water)	0.3	0.1	−0.9	0.1	0.2	−0.1	−0.5	−0.2	−0.5	−0.2
log S (aqueous solubility)	0.03	0.07	−2.0	0.2	−0.7	0.7	0.8	1.2	1.0	0.5

^a^ Solvent accessible surface area (SASA) calculated with a probe of 1.4 Å radius. Drug properties were calculated with the BOSS 4.9 software according to published procedures [35].

**Table 2:** Calculated potential energy of interaction (ΔE) and free energy of hydration (ΔG) for the interaction of fluevirosines and fluevirosinines with the tubulin dimer.

Compounds	ΔE (kcal/mol)	ΔG (kcal/mol)
Securinine	−44.60	−17.10
Norsecurinine	−44.20	−17.10
Fluevirosine A	−83.00	−23.95
Fluevirosine B	−75.00	−27.10
Fluevirosine C	−69.6.0	−23.90
Fluevirosine D	−94.55	−38.80
Fluevirosine G	−89.2.0	−35.85
Fluevirosine H	−86.40	−26.35
Fluevirosinine A	−103.05	−37.80
Fluevirosinine B	−97.35	−34.40
Fluevirosinine C	−97.30	−37.45
Fluevirosinine D	−104.50	−35.40
Fluevirosinine E	−89.45	−35.20
Fluevirosinine F	−102.90	−36.40
Fluevirosinine G	−117.00	−32.90
Fluevirosinine H	−113.90	−45.10
Fluevirosinine I	−102.75	−39.15
Fluevirosinine J	−103.35	−41.10

## Data Availability

The data are contained within the article.
